# Identification of the main malaria vectors in the *Anopheles gambiae *species complex using a TaqMan real-time PCR assay

**DOI:** 10.1186/1475-2875-6-155

**Published:** 2007-11-22

**Authors:** Chris Bass, Martin S Williamson, Craig S Wilding, Martin J Donnelly, Linda M Field

**Affiliations:** 1 Department of Biological Chemistry, Rothamsted Research, Harpenden, AL5 2JQ, UK; 2 Vector Group, Liverpool School of Tropical Medicine, Pembroke Place, Liverpool L35QA, UK

## Abstract

**Background:**

The *Anopheles gambiae sensu lato *species complex comprises seven sibling species of mosquitoes that are morphologically indistinguishable. Rapid identification of the two main species which vector malaria, *Anopheles arabiensis *and *An. gambiae sensu stricto*, from the non-vector species *Anopheles quadriannulatus *is often required as part of vector control programmes. Currently the most widely used method for species identification is a multiplex PCR protocol that targets species specific differences in ribosomal DNA sequences. While this assay has proved to be reasonably robust in many studies, additional steps are required post-PCR making it time consuming. Recently, a high-throughput assay based on TaqMan single nucleotide polymorphism genotyping that detects and discriminates *An. gambiae s.s *and *An. arabiensis *has been reported.

**Methods:**

A new TaqMan assay was developed that distinguishes between the main malaria vectors (*An. arabiensis *and *An. gambiae s.s.*) and the non-vector *An. quadriannulatus *after it was found that the existing TaqMan assay incorrectly identified *An. quadriannulatus*, *An. merus *and *An. melas *as *An. gambiae s.s*. The performance of this new TaqMan assay was compared against the existing TaqMan assay and the standard PCR method in a blind species identification trial of over 450 samples using field collected specimens from a total of 13 countries in Sub-Saharan Africa.

**Results:**

The standard PCR method was found to be specific with a low number of incorrect scores (<1%), however when compared to the TaqMan assays it showed a significantly higher number of failed reactions (15%). Both the new vector-specific TaqMan assay and the exisiting TaqMan showed a very low number of incorrectly identified samples (0 and 0.54%) and failed reactions (1.25% and 2.96%). In tests of analytical sensitivity the new TaqMan assay showed a very low detection threshold and can consequently be used on a single leg from a fresh or silica-dried mosquito without the need to first extract DNA.

**Conclusion:**

This study describes a rapid and sensitive assay that very effectively identifies the two main malaria vectors of the *An. gambiae *species complex from the non-vector sibling species. The method is based on TaqMan SNP genotyping and can be used to screen single legs from dried specimens. In regions where *An. merus/melas/bwambae*, vectors with restricted distributions, are not present it can be used alone to discriminate vector from non-vector or in combination with the Walker TaqMan assay to distinguish *An. arabiensis *and *An. gambiae s.s.*

## Background

The *Anopheles gambiae sensu lato (s.l.) *species complex contains the most important mosquito vectors of malaria in sub-Saharan Africa. It comprises seven morphologically indistinguishable sibling species up to four of which may be sympatric [1-4]. The principal malaria vectors in the complex are *Anopheles gambiae sensu stricto *(s.s.) and *Anopheles arabiensis*. Of the remaining members *Anopheles quadriannulatus *species A, which is widespread in southern Africa, and *Anopheles quadriannulatus *species B, found in Ethiopia, are considered to be zoophilic non-malaria vectors [1,5]. *Anopheles melas *and *Anopheles merus *are both salt water breeding and consequently only important vectors in costal regions [6,7]. The final named member of the complex *Anopheles bwambae *is restricted to a region close to the Buranga hot springs in Uganda [8].

The marked differences in the vectorial efficiency of the species within the complex mean that rapid identification of species is vital for focussed effort in malaria control programmes. To this end a large number of methods have been developed for identification including cross-mating techniques [9], polytene chromosome analysis [2], allozyme electrophoresis [10,11], and gas chromatography of cuticular lipids [12]. However, the most widely adopted method for species identification is based on PCR amplification of ribosomal DNA (rDNA) sequences [13]. The original report of this approach described by Scott *et al *is a multiplex PCR method using species specific primers to amplify products which are of a diagnostic size when visualized by agarose gel electrophoresis. Several modifications or additions to the original method, or similar methods based on the same rDNA sequences have also been reported [3,14-17]. Although the Scott method is now considered to be the 'gold standard' for species identification in this complex recent reports have described non-specific amplification and high rates of failures leading to the need for repeat PCR of the same samples [17]. Additional drawbacks of this method are the time required to process samples when applied to large scale screening of mosquito populations and the safety hazard that ethidium bromide staining of agarose gels entails.

Recently, a high-throughput method for identification of the principal vectors in the complex *An. gambiae *s.s. and *An. arabiensis *has been described [18]. The method is based on TaqMan single nucleotide polymorphism genotyping and represents the first description of a 'closed tube' approach that requires a single step to identify a mosquito DNA sample. The method was tested in a large study of field collected *An. gambiae s.s. *and *An. arabiensis *from western Kenya and was found to have a specificity and sensitivity comparable to standard PCR. However, this assay was not tested using *An. gambiae s.s. *or *An. arabiensis *from other regions in Africa, nor was it tested for its ability to distinguish these two species from the non-vector *An. quadriannulatus*. The current study describes an extensive blind trial of field collected mosquitoes from a range of sites across sub-Saharan Africa comparing the Walker TaqMan SNP genotyping and standard PCR methods with a newly developed TaqMan assay which distinguishes between the principal vector and non-vector species of the complex.

## Methods

### Mosquito samples and preparation of plates for species identification trial

For the initial optimization of each assay, field-caught mosquito specimens from Burkina Faso, Ghana, Kenya, Cameroon and Malawi were used in addition to samples obtained from two laboratory colonies, Kisumu and RSP. These samples included several samples of each of *An. gambiae s.s, An. arabiensis, An. quadriannulatus *species A, *An. melas *and *An. merus*. Samples of the Ethiopian *An. quadriannulatus *species B and *An. bwambae *were not tested in this study because of their more limited distribution.

The blind species identification trials were performed using five 96 well test plates containing 466 samples. Samples were field collected from Cameroon, Ghana, Kenya, South Africa, Malawi, Sao Tome, La Reunion, Tanzania, Sudan, Angola, Burkina Faso, Gabon, and Mozambique. These samples had been initially identified to species at the time of collection using the standard PCR method and included 169 *An. gambiae s.s.*, 173 *An. arabiensis*, 66 *An. quadriannulatus*, and 21 samples of *An. melas *and *An. merus*, the remaining samples were either undetermined or negative controls. This information was withheld from the persons who carried out the testing of each assay to ensure no bias occurred in the scoring of results. For all samples DNA was extracted from single mosquitoes using either the Livak or Ballinger Crabtree methods [19,20]; DNAzol reagent (Molecular Research Centre, Inc) at one-fifth the recommended reagent volume for each extraction or using a crude boil and centrifugation protocol. This was a variant of the STE method of O'Neill *et al*. [21] in which samples were ground in STE buffer (100 mM NaCl, 10 mM Tris-HCl, pH8.0, 1 mM EDTA, pH8.0), heated to 95°C for 5 mins, centrifuged for 3 mins at 13,000 rpm and the supernatant used directly as PCR template. The DNAs were resuspended in either TE buffer or sterile water at volumes between 100 and 200 μl. To determine the sensitivity of the three identification methods a dilution series of the DNA from each of the five main species in the *Anopheles gambiae *complex was included in the trial. For this, DNA preparations were diluted to 20 ng/ul (as determined by absorption at 260 nm using a NanoDrop spectrophotometer, NanoDrop Technologies). The samples were then serially diluted down to a 1 in 1 × 0^6 ^dilution.

### Standard PCR

Standard PCR was carried out as described previously [13].

### Walker TaqMan Assay

The design of a TaqMan assay to distinguish *An. gambiae s.s. *from *An. arabiensis *has been described previously [18]. The PCR conditions used in the study by Walker *et al *are not given in the original manuscript but were obtained from Edward Walker (personal communication). These were modified slightly and were as follows: PCR reactions (25 μl) contained 1 μl of genomic DNA, 12.5 μl of SensiMix DNA kit (Quantace), 900 nM of each primer and 200 nM of each probe. Samples were run on a Rotor-Gene 6000™ (Corbett Research) using the temperature cycling conditions of: 10 minutes at 95°C followed by 40 cycles of 95°C for 20 seconds and 60°C for 45 seconds. The increase in VIC and FAM fluorescence was monitored in real time by acquiring each cycle on the yellow (530 nm excitation and 555 nm emission) and green channel (470 nm excitation and 510 emission) of the Rotor-Gene respectively.

### Novel TaqMan Assay

A novel TaqMan assay that would distinguish the main malaria vectors *An. arabiensis *and *An. gambiae s.s. *from the non-vector *An. quadriannulatus *was designed after it was found the Walker TaqMan assay incorrectly identified *An. quadriannulatus, An. merus *and *An. melas *as *An. gambiae s.s*. An alternative region within the rRNA gene at the 5' end of the intergenic spacer (bases 475 to 487) was selected where the nucleotide sequence in *An. gambiae s.s. *and *An. arabiensis *is GCTCGTCTTGGTC. In *An. quadriannulatus*, *An. melas *and *An. merus *the same region of sequence shows a SNP (GC**G**CGTCTTGGTC). Flanking this SNP was an area of conserved sequence between the species which allowed for the design of forward, comF (5'-GCTTGGTGGTTTGTCCG-3'), and reverse, comR (5'-CTGTGTCGACGTGGTCCC-3'), primers. Antisense minor groove binding (MGB) probes (Applied Biosystems) that bind over the SNP site were designed using the Primer Express™ Software Version 2.0. The probe AG/AA (5'-GACCAAGACGAGC-3') was labelled with 6-FAM at the 5' end for the detection of *An. gambiae s.s. *and *An arabiensis *and the probe AQ/AM (5'-GACCAAGACGCGC-3') was labelled with VIC for detection of *An. quadriannulatus*, *An. melas *and *An. merus*. Each probe also carried a 3' nonfluorescent quencher and a minor groove binder at the 3' end. The minor groove binder provides more accurate allelic discrimination by increasing the T_M _between matched and mis-matched probes [22]. This new TaqMan assay was used in the blind genotyping trial to score samples into a group containing the principal malaria vectors in the complex *An. gambiae s.s. *and *An. arabiensis *and a second group containing *An. quadriannulatus, An melas *and *An. merus*. Samples that had scored as 'vectors' were then further identified to species using the Walker TaqMan assay.

PCR reactions (25 μl) contained 1 μl of genomic DNA, 12.5 μl of SensiMix DNA kit (Quantace), 800 nM of each primer and 200 nM of each probe. Samples were run on a Rotor-Gene 6000™ (Corbett Research) using the temperature cycling conditions of: 10 minutes at 95°C followed by 45 cycles of 95°C for 15 seconds, 50°C for 20 seconds and 72°C for 20 seconds. The increase in VIC and FAM fluorescence was monitored in real time by acquiring each cycle on the yellow (530 nm excitation and 555 nm emission) and green channel (470 nm excitation and 510 emission) of the Rotor-Gene respectively. PCR reactions were also carried out using whole mosquitoes or single legs instead of genomic DNA, in this case the leg or body was simply placed in the PCR tube and covered with the PCR mastermix.

## Results

### Standard PCR

**Figure 1:**
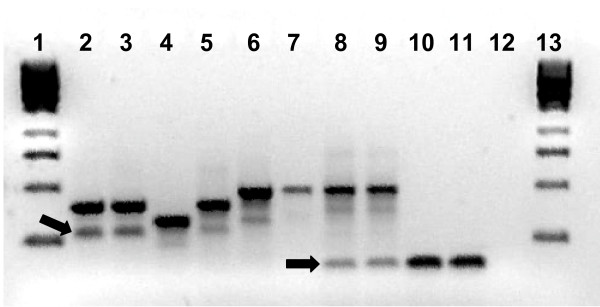
**Species identification using the standard PCR method of Scott et al**. The arrows highlight the presence of non-specific bands. Lanes contain (1,13) 1 kb DNA marker, (2,3,5) *An. gambiae s.s*., (4) *An. arabiensis*, (6,7), *An. melas *(8,9), *An. merus *and (10,11) *An. quadriannulatus*, (12) no template control.

After minimal optimization the multiplex PCR method designed by Scott *et al *was found to effectively identify control templates of known species. As reported previously, agarose gel electrophoresis of PCR products did show the presence of non-specific bands in some species (Figure 1) and in some instances these were of the same size as diagnostic bands which made scoring genotypes less straightforward. An example of this (Figure 1) is the small non-specific band sometimes produced in *An. merus *samples which is of the same size as the diagnostic band seen in *An. quadriannulatus*. As shown in Figure 1, the standard PCR method does not discriminate between *An. melas *and *An. merus *samples as these produce diagnostic fragments of 464 bp and 466 bp in size respectively. Samples producing fragments of this size were therefore scored as *An. melas/An. merus *in the genotyping trial. The results from the blind species identification trial using the PCR method are shown in Table 1. The standard PCR method showed good analytical specificity with only three incorrect identifications (0.75%). Two of these were incorrectly scored as *An. quadriannulatus *and *An. gambiae s.s. *hybrids which may be due to the non-specific amplification described previously. In this trial the PCR method showed a reasonably high PCR failure rate of 15% (60 failed reactions not including the dilution series). The analytical sensitivity of the PCR method was examined using a dilution series of DNA of each of the five species, both as part of the blind trial and also subsequently with dilutions of two additional DNA templates of each species to check for variation between templates. Sensitivity varied slightly depending on species but in general the limit of detection was between a 1 in 100 to a 1 in 200 dilution representing 0.1–0.2 ngs of DNA in PCR except for *An. quadriannulatus *where the sensitivity was reduced to a 1 in 10 dilution or 2 ng of DNA in PCR.

**Table 1:** Performance of three assays in the blind species identification trial.

	**Genotyping results**		
	**new TaqMan**	**AS PCR**	**Walker TaqMan**
Correct scores	455/466	364/466	333/371
Failed reactions	11	99	25
Failed reactions			
excluding dilution series	5	60	11
Incorrect scores	0	3	2

### Walker TaqMan Assay

**Figure 2:**
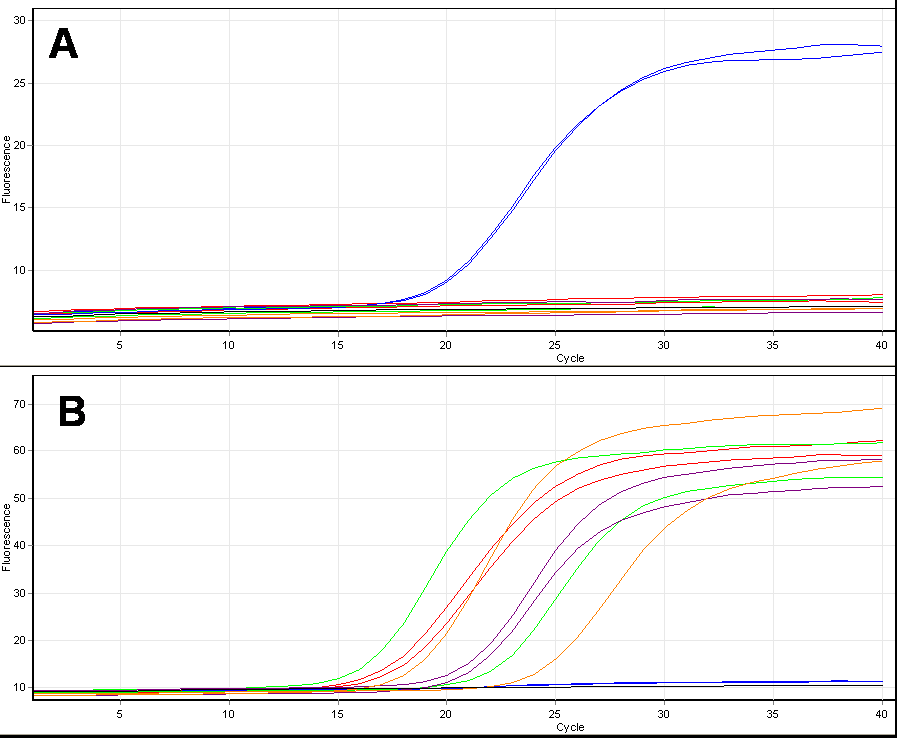
**Species identification using the real-time TaqMan assay designed by Walker et al**. In this example two specimens of *An. gambiae s.s*., (red trace) *An. arabiensis*, (blue trace) *An. melas *(green trace), *An. merus *(purple trace) and *An. quadriannulatus *(orange trace) were tested. Part A displays the cycling of FAM-labelled probe specific for *An. arabiensis*. Part B displays the cycling of the VIC-labelled probe designed to be specific for *An. gambiae s.s. *and shows the non-specific amplification of the *An. melas*, *An. merus *and *An. quadriannulatus *samples.

The TaqMan assay designed by Walker *et al *for detection of *An. gambiae *s.s. and *An. arabiensis *was initially optimized using control templates of known species. This showed that the assay successfully identifies the two target species as expected. However, when the assay was tested with other species in the complex it was found that *An. quadriannulatus*, *An. melas *and *An. merus *are also detected by the VIC labelled probe, designed to detect *An. gambiae s.s*., leading to incorrect scoring of these species (see Figure 2). Attempts were made to further optimize the assay and increase the specificity of the VIC probe by altering annealing temperature and magnesium chloride concentration but significant improvements in specificity were not obtained. After trialling the assay with several templates of *An. quadriannulatus*, *An. melas *and *An. merus *to check that the non-specific detection was consistent, the assay was used in the blind species identification trial only for templates that had been scored previously as *An. gambiae s.s./An. arabiensis *using the newly developed TaqMan assay (see below). The results using this reduced number of 371 (Table 1) showed a low assay failure rate of ca. 3% (11 failed reactions) and excellent analytical specificity on the diverse collection of *An. gambiae s.s. *and *An. arabiensis *samples included in the trial with only 2 incorrect identifications (0.54%). In both instances these incorrect identifications were specimens scored as *An. gambiae s.s./An. arabiensis *hybrids, this low error rate (ca. 1%) in mistakenly identifying species hybrids was also reported by Walker *et al*. The analytical sensitivity of the PCR method was examined in a dilution series of DNA of each of *An. gambiae s.s. *and *An. arabiensis *as part of the blind trial and also subsequently with dilutions of two additional DNA templates. The limit of detection for each species was between 1 in 1600 and 1 in 10 000 dilution representing 2–12.5 picograms of DNA in PCR for *An. gambiae s.s. *and between a 1 in 100 and 1 in 400 dilution 50–200 picograms of DNA in PCR for *An. arabiensis*.

### Novel TaqMan Assay

**Figure 3:**
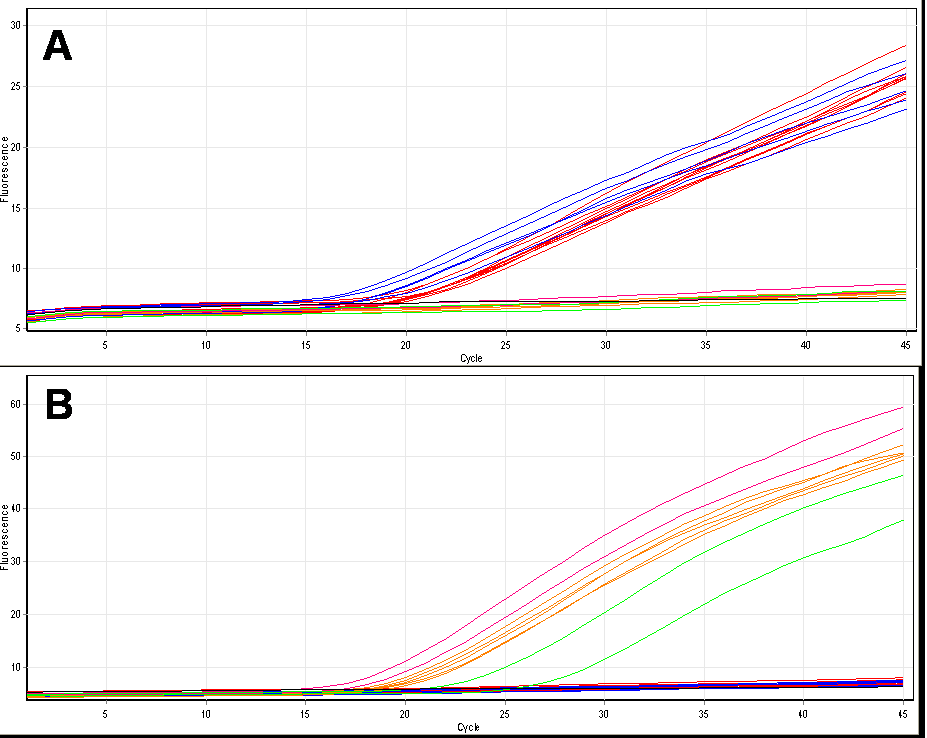
**Species identification using the newly developed TaqMan assay designed to distinguish the principal vector species *An. gambiae s.s. *and *An. arabiensis *from the other members of the complex**. In this example two or more specimens of *An. gambiae s.s*., (red trace) *An. arabiensis*, (blue trace) *An. melas *(green trace), *An. merus *(purple trace) and *An. quadriannulatus *(orange trace) were tested. Part A displays the cycling of FAM-labelled probe specific to *An. arabiensis *and *An. gambiae s.s*. Part B displays the cycling of the VIC-labelled probe specific to *An. quadriannulatus, An. melas *and *An. merus*.

After optimization the TaqMan assay developed in this study effectively identified control templates of known species. This assay uses two probes, the first labelled with 6FAM will detect both the principal malaria vectors in the complex *An. gambiae s.s. *and *An. arabiensis*, and the second probe, labelled with VIC, will detect *An. quadriannulatus*, *An. melas *and *An. merus*. Thus, a substantial increase in FAM fluorescence during PCR indicates an *An. gambiae s.s. *or *An. arabiensis *specimen while a substantial increase in VIC fluorescence indicates an *An. quadriannulatus*, *An. melas *or *An. merus *specimen (Figure 3). An increase in both dyes would indicate a hybrid or a contaminated sample. The ability of this assay to differentiate between the principal malaria vectors and the non-vector *An. quadriannulatus *is the significant advantage of this method to the Walker TaqMan.

**Figure 4:**
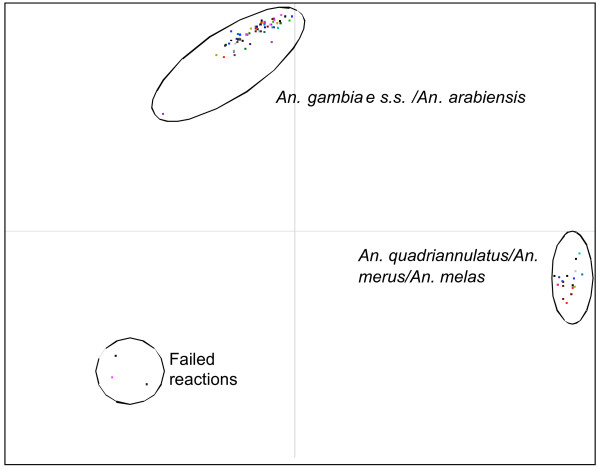
**Scatter plot analysis of TaqMan fluorescence data**. In this example real time PCR was carried out using the newly developed TaqMan assay designed to distinguish the principal vector species *An. gambiae s.s. *and *An. arabiensis *from the other members of the complex on 96 samples. Fluorescence values of the FAM labelled probe specific for *An. gambiae s.s. *and *An. arabiensis *were then plotted against the VIC labelled probe specific for *An. quadriannulatus, An. melas *and *An. merus*.

To help identify species the Rotor-Gene software allows endpoint fluorescence values for the two dyes to be automatically corrected for background and plotted against each other in bi-directional scatter plots (Figure 4). The clustering of samples in scatter plots in addition to the real-time fluorescence traces gives easy, objective and accurate species scoring. The results of using this method in the blind species identification trial are shown in Table 1. The new TaqMan showed excellent analytical specificity with no incorrect identifications, it also showed the lowest level of failed samples of the three assays trialled with 5 failed reactions (1.25%). This low rate of failure was probably due to the high analytical sensitivity of this assay which was examined in a dilution series of DNA of each of the five species both as part of the blind trial and also subsequently with dilutions of two additional DNA templates. The limits of detection were around a 1 in 100 000 dilution or 0.2 picograms of DNA in PCR and in one of the experiments a 1 in 1000 000 dilution or 20 femtograms of DNA in PCR was detected. Because of this high degree of sensitivity we carried out additional experiments to investigate the possibility of using whole mosquitoes or single mosquito legs in PCR instead of genomic DNA. In this case, the leg or body was simply placed in the PCR tube and covered with the PCR reaction mix. When whole mosquito bodies were used as template for PCR we found a high level of background fluorescence which prevented clear scoring but using single legs from fresh or silica dried mosquitoes was found to work nearly as well as using extracted DNA.

## Discussion

Identification of the species in the *Anopheles gambiae *complex is important for ecological research studies interested in the geographic distribution, abundance and behaviour of the different vector species. In addition as the complex comprises species with different efficiencies as vectors, and includes non-vector species accurate identification is also paramount for vector control programmes. In this blind species identification trial of three assays using a range of mosquito specimens collected from a wide variety of geographic areas the most widely used multiplex PCR method was compared with two TaqMan assays, one which has been described previously and one which is described here for the first time.

The standard PCR method was found to be very specific with a low rate of incorrect scores (<1%), however, when compared to the TaqMan assays it showed a significantly higher rate of failed reactions (15% compared to 1.25% and 2.96%). This was noticed to occur more frequently using DNA templates that had been extracted by the STE buffer protocol which, in the authors' experience, is quick to carry out but yields DNA that is of lower quality and more likely to degrade with time or multiple freeze-thawing. The chief disadvantage of this method is the requirement for the post-PCR processing of samples by agarose gel electrophoresis. This is time consuming, restricts throughput, requires the use of the safety hazard ethidium bromide, and variation in the quality of the agarose gels can lead to difficulties in interpreting results.

The TaqMan method, in contrast to standard PCR, does not require post-PCR processing due to the real-time detection of the specific binding of fluorescently labelled probes during PCR. This 'closed-tube' approach makes the method high-throughput and simple to carry out. Initially, a recently developed TaqMan method that identifies the two main mosquito vector species of the complex *An. gambiae *s.s. and *An. arabiensis *was trialled [18]. During optimization with mosquito samples of known species it became clear that this assay could not be used to differentiate these two members of the complex from the others as *An. melas, An. merus *and the non-vector species *An. quadriannulatus *were all incorrectly identified as *An. gambiae s.s*. Closer examination of the 5' intergenic spacer region of rDNA to which the probes bind reveals that while *An. gambiae s.s. *and *An. arabiensis *differ at seven positions *An. melas *and *An. merus *differ from *An. gambiae s.s. *at only two positions and *An. quadriannulatus *at only one which may explain the non-specific amplification exhibited. If this method was able to accurately identify *An. gambiae s.s. *and *An. arabiensis *from the other members of the complex one additional potential disadvantage of the assay design is that an *An. gambiae s.s. *or *An. arabiensis *specimen that failed in the PCR reaction might be incorrectly scored as *An. melas*/*An. merus*/*An. quadriannulatus *as these would also score as 'no templates/fails'.

The TaqMan assay developed in this study is designed to distinguish between the main malaria vectors *An. gambiae s.s. *and *An. arabiensis *as one group and *An. quadriannulatus, An. melas *or *An. merus *as a second group. The rationale behind this design is that although *An. melas *and *An. merus *have been shown to be malaria vectors their distribution is limited to coastal regions as a consequence of their requirement for brackish water to breed. In contrast, *An. quadriannulatus *species A which is a non-vector species is widespread in southern Africa and is sympatric in many areas with *An. gambiae s.s. *and *An. arabiensis*. Therefore, there is a considerable need in many vector control programs to rapidly distinguish between the two main vector species and the non-vector species. The new TaqMan addresses this requirement and has application as a vector/non-vector identification test for large parts of sub-Saharan Africa. An alternative application for this assay is to distinguish *An. melas *from *An. gambiae s.s./An. arabiensis *where they are sympatric such as regions along the west coast of Africa. This is viable as the former does not occur sympatrically with *An. merus *or *An. quadriannulatus *[5]. It should, however, be used with caution in regions close to the east coast of Africa where *An. quadriannulatus *has been found to occur sympatrically with *An. merus *[5]. In contrast to the previous TaqMan assay this method will also correctly identify a failed reaction as all members of the complex (except for *An. bwambae *which was not tested due to its rarity) are detected by one of the two fluorescently labelled probes. Used alone the new TaqMan assay will not distinguish between *An. gambiae s.s. *and *An. arabiensis *which may be significant for certain studies, such as those examining the spread of resistance genes in mosquito populations. However if the identification of *An. gambiae s.s. *from *An. arabiensis *is required, then the two TaqMan assays described here can be used sequentially. In this case the new TaqMan is run first having the advantage that it can be used on a single leg from a silica-dried mosquito without the need to first extract DNA. Samples that are scored as *An. gambiae s.s.*/*An. arabiensis *can then be further identified to species using the Walker TaqMan. This approach was used in the blind species identification trial and proved to be successful, taking less time to genotype all samples than the standard PCR method. It may be possible in future to increase throughput and reduce consumable costs by combining the two TaqMan assays so that *An. gambiae s.s, An. arabiensis *and *An. quadriannulatus *can be detected in a single tube using probes labelled with fluorophores with distinct emission and excitation spectra. An additional advantage of the TaqMan assays seen in the species identification trial was the very low rate of incorrectly identified samples (0 and 0.54%) and failed reactions (1.25% and 2.96%) which again increases throughput over the standard PCR as it alleviates the need to spend time repeating reactions.

## Conclusion

In a trial of two TaqMan assays with standard PCR for the identification of members of the *Anopheles gambiae *species complex the TaqMan assays were higher throughput, more sensitive and less prone to failure of amplification than standard PCR.

## Authors' contributions

CB designed the study, developed the new TaqMan assay, optimized the standard PCR method and the previously described TaqMan method and drafted the manuscript. CSW and MJD collected mosquito specimens, extracted DNA and helped draft the manuscript. MSW helped design the study, prepared the plates of mosquito DNAs and helped draft the manuscript. LMF helped design the study and helped draft the manuscript. All authors read and approved the final manuscript.
